# Does obesity create a relative sense of excess poverty?

**DOI:** 10.3389/fpubh.2024.1480365

**Published:** 2024-11-27

**Authors:** Yuval Arbel, Yifat Arbel, Amichai Kerner, Miryam Kerner

**Affiliations:** ^1^Sir Harry Solomon School of Economics and Management, Western Galilee College, Acre, Israel; ^2^Department of Mathematics, Bar Ilan University, Ramat Gan, Israel; ^3^Faculty of Social Sciences, Banking and Finance Program, Bar Ilan University, Ramat Gan, Israel; ^4^The Ruth and Bruce Rapoport Faculty of Medicine, Technion – Israel Institute of Technology, Haifa, Israel; ^5^Department of Dermatology, Emek Medical Center, Afula, Israel

**Keywords:** obesity, poverty, public health, a follow-up survey, lagged variables

## Abstract

**Background:**

This study investigates the potential relationship between obesity and self-ranking of poverty, as a proxy for self-awareness and happiness. To the best of our knowledge, this issue has not been previously explored based on self-ranking of poverty when income is controlled.

**Method:**

Ordered Probit Regressions. We propose a new measure for the influence of western social values and norms associated with discrimination against obese women.

**Results:**

Based on a follow-up survey after two years, findings demonstrate a *drop* in the projected probability of self-ranking as “not poor” with the *BMI* from 0.73 to 0.37 (females) – 0.48 (males) when the level of income is controlled. Similar outcomes are obtained when the independent variables are lagged and thus avoid endogeneity concerns. Finally, additional outcomes support the conclusion that the lagged *BMI* Granger-cause self-ranking of poverty for women, but not for men. Findings support the awareness of more obese women to lower prospects of finding a job.

**Conclusion:**

Since according to twin studies, approximately 80% of obesity emanates from genetic factors, research findings stress the need to educate the public against prejudices on the grounds of obesity. In particular, our study seeks to evoke awareness among potential employers, which, in turn, might motivate avoidance of, or at least reduction in, an implicit wage penalty against obese women.

## 1 Introduction

Obesity is a global epidemic and a risk factor for non-communicable diseases and mortality. More than half of the population in 34 of the OECD countries are currently overweight, whereas one in four is obese. From 2010 to 2016, there was a 3 % increase in the number of people suffering from obesity, i.e., – 50 million more people in the OECD countries.

One of the measures for the damages associated with obesity is the lost years of life (1). Among the OECD countries, the life span in Mexico is shortened by 4.2 years, in Poland and Russia by 3.9 years, in the USA and Hungary 3.7 years, in Romania 3.5 years, in Israel—3.4 years, in Ireland 2.9 years, in France 2.7, in South Korea 1.7 and in Indonesia 1.5 years due to obesity. The list is closed by Japan – where obesity shortens life by only one year. Diseases associated with obesity will claim the lives of approximately 90 million people in OECD countries over the next 30 years.

Education and socio-economic background affect obesity. Reciprocally, obesity damages labor market outcomes that, in turn, contribute to reinforcing existing social inequalities (2). Obese people have poorer job prospects compared to normal-weight people, they are less likely to be employed and have more difficulty re-entering the labor market (3). Obese people are less productive at work due to more sick days and fewer worked hours, and they earn about 10% less than non-obese people. Addressing obesity and the associated negative labor market outcomes would help break the vicious circle of social and health inequalities.

Indeed, numerous studies have demonstrated the positive relationship between poverty and obesity. Yet, the extent to which obese people have an increased perception of being poor when the actual level of income is controlled remains an open question.

Previous studies have revealed the stigmatization of obese persons by society. Obese people have frequently been found to use language, which reflects poor self-identity following the perceived negative impact of their weight (4). Likewise, obese persons seem to suffer more from poor self-esteem, as well as a higher level of vulnerability and a propensity to depression, particularly among women (5).

Previous studies have also demonstrated wage and other penalties against obese people – particularly women (6–14).

Yet, with one exception (8); the question that remained open is the self-awareness of obese women and men to these penalties. Our study contributes by demonstrating this awareness while the income level is controlled. They support the awareness of more obese women, manifested by their subjective ranking as “not poor,” to lower prospects of finding a job.

The objective of the current study is to demonstrate that obese people suffer not only from poor self-esteem, but also from increased sense of subjective poverty among both genders, even when the actual level of income is controlled. The rationale of our study is the following. *A-priori*, compared to normal weight persons there is no reason that obese persons would have a sense of excess poverty where the level of income is controlled. Yet our study is the first to clearly demonstrate elevated awareness to weight discrimination among obese persons.

Indeed, many studies in the literature demonstrate wage discrimination against obese persons and particularly women. Based on panel setting, Caliendo and Gehrsitz (12) suggest that for a 1-point *BMI* increase in Germany, wage drops by 0.6–0.7% among women both in blue and white-collar professions (page 216). The authors mention the robust findings in the literature that unlike women, men are either not subject to weight penalties or premia in the labor market, or at least experience a diminished wage penalty [e.g., (15, 16)]. Campos-Vazquez and Gonzalez (17) show lower prospects of finding a job among obese women in Mexico, but not among obese men. Finally, Prioschery et al. (18) demonstrate the importance of western values referring to the body silhouettes and obesity of South African urban females.

Puhl and Brownell (19) argue that discrimination against obese persons can be documented in three important areas of living: employment, education, and health care. 28% of teachers in one study claimed that becoming obese is the worst thing that can happen to a person; controlling for income and grades, parents provide less college support for their overweight children than for their “thinner” children; 24% of nurses said that they are “repulsed” by obese persons.

Finally, Böckerman et al. (20), suggest that the outcomes obtained from economic models using the narrower genetic risk score as an instrument indicate 6.9% lower wages, 1.8% fewer years employed, and a 3-percentage point higher probability of receiving any social income transfers following a one-unit increase in BMI in Finland.^1^ Note, however, that unlike Böckerman et al. (20), who discusses the impact of the genetic profile, the current paper discusses the socio-cultural factor, namely, the impact of the social stigma on the subjective sense of poverty among obese persons.

We propose a new measure for the influence of western socio-cultural values and norms associated with discrimination against obese women. Given the lower prospects among these women of finding a job, one would anticipate a positive relationship between obesity (represented by higher body-mass index *BMI* $=\frac{\mathrm{kg}.}{\mathrm{mete}r^{2}}$ ) (where weight is measured in kilograms and height is measured in meters) and higher ranking of poverty.^2^

This study is based on the longitudinal survey carried out by the Israeli Central Bureau of Statistics (ICBS) (21) and based on a representative sample of the Israeli population. The survey reports the response of interviewers to the following question: “During the last 15 years, how often did you consider yourself poor?” on a scale of 1 = “often” to 4 = “never,” as well as an objective measure of income [total gross annual income from all sources in NIS (the local Israeli currency, where 1 NIS ≈ $0.31)]. Additional recorded information is the weight and height of each individual, from which the *BMI* measure may be calculated.

Findings clearly demonstrate a *drop* in the projected probability of self-ranking as “not poor” with *BMI* ranging from 0.73 to 0.37 (females) – 0.48 (males) when income is controlled. Similar outcomes are obtained when the independent variables are lagged and thus avoid endogeneity concerns. Finally, additional outcomes support the conclusion that the lagged *BMI* Granger-cause self-ranking of poverty for women, but not for men. Consequently, the outcomes of our study demonstrate awareness to the economic outcomes of discriminations against obese persons. These phenomena are plausible given the lower frequency of dates and jobs opportunities (17, 22), which, in turn, diminishes the Social and Economic Status (SES) in the long run.

The contribution of this study lies in its focus on economic parameters. The focus of previous studies was obesity as a precursor of lack of self-confidence, as well as increased depression and vulnerability (4, 22), or the genetic component of obesity as a precursor of lower wages, fewer years employed, and a higher probability of receiving any social income transfers (20). Yet, to the best of our knowledge, the current study is the first to measure the subjective sense of poverty among obese persons (i.e., how often you consider yourself poor) when the level of income is controlled. Thus, the outcomes of our study can be interpreted as the degree of awareness of the social stigma concerning obese persons among potential employers, parents, and mates. In this context, a recent article by Campos-Vazquez & Gonzalez (17) indeed demonstrated a lower prevalence of job offers to obese women – where all the other C.V. factors were controlled.

In sum, the contribution of this research lies in the new method proposed to assess the permanent income of obese persons. According to Friedman (23) the permanent income hypothesis is a theory of consumer spending stating that people will spend money at a level consistent with their expected long-term average income. The outcomes of the current study indicate that the level of permanent income among obese people is lower than their current income.

According to twin studies, approximately 80% of obesity emanates from genetic factors (24). Consequently, research findings stress the need to educate the public against prejudices on the grounds of obesity. In particular, our study seeks to evoke awareness among potential employers, which, in turn, might motivate avoidance of, or at least reduction in, an implicit wage penalty against obese women.

The implication of twin studies is the comparison between identical (monozygotic) twins. This is a conventional methodology in medical literature, particularly where the indication of genetic disorder emerges. In research, concordance is often discussed in the context of both members of a pair of twins. Twins are concordant when both have, or both lack a given trait.

One example is Ji and An (25). Using the twin study design, and subsequent control for genetics and shared environmental effects, the authors found negative association between harsher parenting in communication and BMI in German twin families. Another example is Lietzén et al. (26), who studied the effects of regular exercise training on LFC, PFC, and metabolism in monozygotic twin pairs discordant for BMI.^3^

The remainder of this article is organized as follows. Section 2 provides the description of data and methods. Section 3 gives the results. Finally, section 4 provides discussion and section 5 concludes and summarizes.

## 2 Data and methods

### 2.1 Description of data

The data are obtained from the 2015 and 2016 waves of a longitudinal survey carried out by the Israeli Central Bureau of Statistics (ICBS) (21). Given the conduct of the survey by ICBS – a government agency – supervised by the Organization of Cooperation and Economic Development (OECD), it is evident that rigorous measures were undertaken to ensure that the 2015 baseline is a representative sample of the Israeli population. A big advantage of governments authorities is their potential ability to enforce cooperation of the individuals randomly assigned to participate in the survey. In fact, many macro level outcomes reported as part of the national accounting of Israel are based on this sample rather than the whole population.

Within the framework of the survey, interviewers returned in 2016 to the same participants in 2015 and asked them the same questions. In the basic results sub-section only the 2016 wave is employed and analyzed. As a robustness test, and as explained below, in the robustness test sub-section, data from both waves are used, where the empirical model is based on lagged independent variables. This methodology prevents or reduces endogeneity concerns.^4^

### 2.2 Descriptive statistics

Table 1 provides the descriptive statistics of the variables, which are later incorporated into the empirical model, and refers to the 2016 wave of the survey. While the *Self_Rank_Poverty_*2016 variable gives the subjective measure of poverty (self-ranking of poverty in response to the question: “During the last 15 years, how often did you consider yourself poor?” on a scale of 1 = “often” to 4 = “never”), the total_inc2016 variable provides the objective proxy of income level (total gross annual income from all sources in NIS).

**Table 1:** Descriptive statistics – 2016 wave of ICBS longitudinal survey.

A. Description of variables	
Variable	Description
*Self_Rank_Poverty_*2016	Self-ranking of individuals in response to the question: “During the last 15 years, how often did you consider yourself poor?” in 2016. Possible answers are 1 = “often,” 2 = “sometimes,” 3 = “rarely,” 4 = “never.”
Total_inc2016	Total gross annual income from all sources in NIS
BMI2016	Body mass index= $\frac{\mathrm{kg}.}{\mathrm{mete}r^{2}}$ where 18 ≤ BMI ≤ 25 is normal weight; 25 ≤ BMI < 30 is overweight; BMI ≥ 30 is considered obesity
Female2016	1 = female; 0 = male

B. Descriptive statistics (unweighted means)				
Variable (*N* = 4,017)	Mean	Std. Dev.	Pct	Median
*Self_Rank_Poverty_*2016	3.295	1.112	100.00%	4.000
Often			14.190%	
Sometimes			8.890%	
Rarely			10.130%	
Never			66.790%	
Total			100.00%	
Total_inc2016	121,721 [117,867,125,574]	124,569		84,969
BMI2016	25.616	4.272		24.880
Gender				
Female2016			44.511%	0
Male2016			55.489%	
Total			100.00%	

C. Descriptive statistics (weighted means)			
Variable (*N* = 4,017)	Mean	Linearized standard error	95% CI
*Self_Rank_Poverty_*2016	3.279	0.003	[3.273, 3.285]
Total_inc2016	122,507.2	1,959.858	[118,664.800, 126,349.600]
BMI2016	26.329	0.077	[26.178, 26.479]

The table refers to the 2016 wave of the longitudinal survey carried out by the ICBS. The survey data analysis includes four strata based on the four categories of the variable *Self_Rank_Poverty_2016*. The weight given to each observation is based on the inverse of the BMI variable. Based on the analysis the population size is 102,900.72. 95% confidence intervals are given in square parentheses.

Referring to the ordinal *Self_Rank_Poverty_*2016 variable 14.19% of the respondents noted that they often considered themselves poor and 66.79% noted that they never considered themselves poor. These responses cover 80.98% of the sample of 4,017 subjects. The implication is left-skewed distribution of *Self_Rank_Poverty_*2016, which can also be inferred from the fact that the median of the *Self_Rank_Poverty_*2016 variable (=4) is higher than the mean (=3.295).

Referring to the total_inc2016 variable, the sample median is 84,969 NIS and the sample mean is 121,721 NIS. Given that the sample mean, affected by outliers, is greater than the sample median, the implication is right-skewed distribution. According to the ICBS (27) press release from May 7, 2017, the gross monthly wage in December 2016 is 10,123 NIS, and the equivalent annual wage is 121,476 NIS. Based on the 95% confidence interval in the sample [117,867–125,574], one cannot reject the null hypothesis that the sample mean equals to that of the population mean in December 2016.^5^

**Figure 1:**
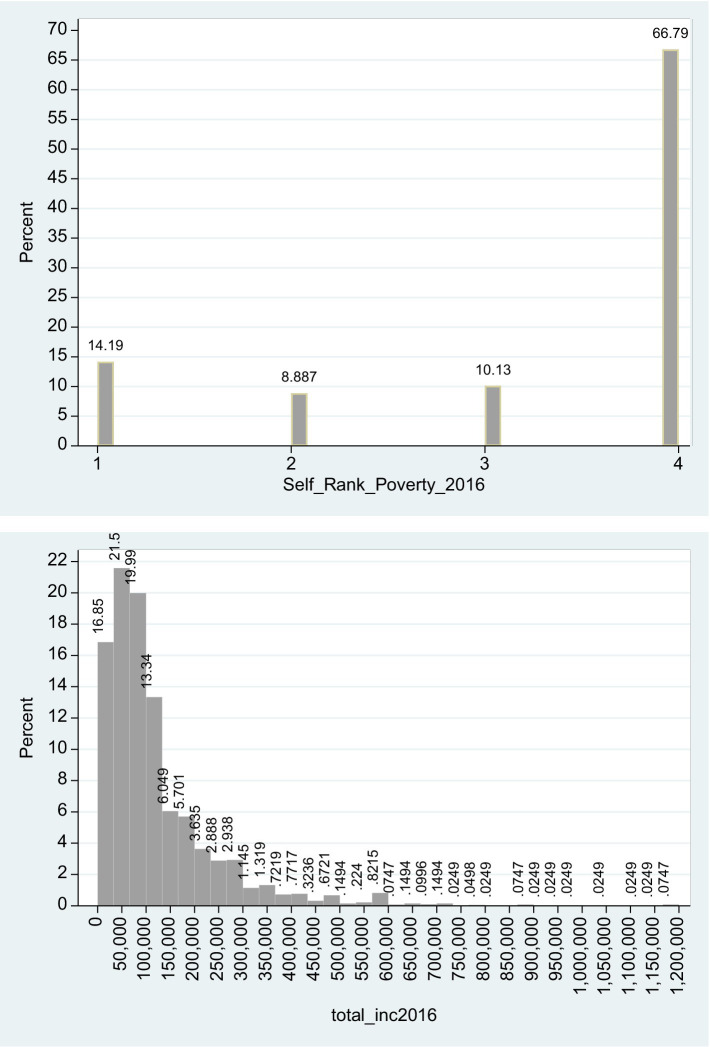
Histograms of self-ranked poverty and actual income in 2016. The histograms refer to 4,017 subjects. The skewness of *Self_Rank_Poverty_*2016 (total_inc2016) is −1.219 (+2.895) and for both variables the null hypothesis of symmetrical distribution (zero skewness) is clearly rejected.

Figure 1 describes the histograms of the variables *Self_Rank_Poverty_*2016 and total_inc2016. Indeed, as can be seen from Figure 1, while the distribution of 2016 poverty ranking is left-skewed, the distribution of 2016 total income from all sources is right-skewed. The implication is consistent with the findings of Stessman et al. (28), who demonstrated that in the older population above 70 years there is no overlapping between subjective and objective poverty. While the latter is measured based on the poverty line (below median net income),^6^ the former is based on the subjective feeling of the individual that the net income is insufficient to cover monthly expenses. Moreover, higher levels of subjective poverty are associated with higher levels of depression and low self-assessment of health conditions (28).

### 2.3 Method

The conventional way to describe the empirical model is the following:


$$\begin{array}{l} \mathrm{Self}\_\mathrm{Rank}\_\mathrm{Poverty}\_2016=\alpha_{1}\ln\left(\mathrm{Total}\_Inc2016\right)\\ +\alpha_{2}BMI2016+\alpha_{3}+\in \end{array}$$

Where the dependent variable Self_Rank_Poverty_2016 is the self-ranking of individuals in response to the question: “During the last 15 years, how often did you consider yourself poor?” Possible answers are 1 = “often,” 2 = “sometimes,” 3 = “rarely,” 4 = “never”; the independent variables are $\ln\left(\mathrm{Total}\_Inc2016\right)$ and BMI2016 .^7^ $\alpha_{1},\alpha_{2},\alpha_{3}$ are parameters and ϵ is the classical random disturbance term.

One concern referring to the conventional model is the fact that the dependent variable Self_Rank_Poverty_2016 is ordinal, namely, the scale 1, 2, 3 and 4 has no definite quantitative interpretation. One could argue that a different scale could be employed. To address this concern, we use instead the ordered probit regression (for a detailed description see Appendix A). The estimation of the model yields projected probabilities of the choice Self_Rank_Poverty_2016=1,2,3,4 as a function of the two independent variables Total_Inc2016 and BMi2016 .

This model is well established in empirical literature [e.g., (29, 30)]. As Frey and Stutzer (29) argue: “Provided that reported subjective well-being is a valid and empirically adequate measure for human well-being, it can be modeled in a microeconometric happiness function $W_{\mathrm{it}}=\alpha +\beta X_{\mathrm{it}}+\epsilon_{\mathrm{it}}$ that is estimated by ordered probit or logit” (page 406).

**Figure 2:**
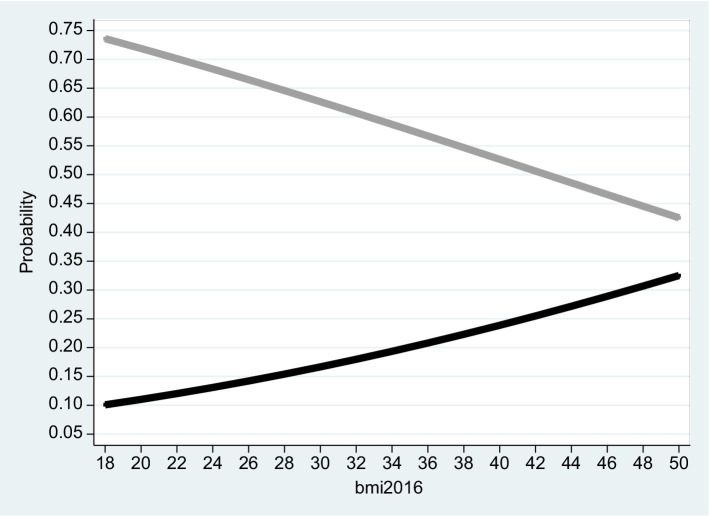
Pooled sample 2016. The figure is based on projected probabilities to report: “I often consider myself poor” (black line) and “I never consider myself poor” (grey line) as a function of the BMI level. The transformations to these projected probabilities are based on the outcomes reported on column (1) in Table 2. The horizontal axis is the BMI level on a scale of between 18 to 50. *BMI* (= $\frac{\mathrm{kg}.}{\mathrm{mete}r^{2}}\alpha$ where 18 ≤ *BMI* ≤ 25 is normal weight; 25 ≤ *BMI* < 30 is overweight; *BMI* ≥ 30 is considered obesity – see the links to the WHO websites in the reference list).

**Figure 3:**
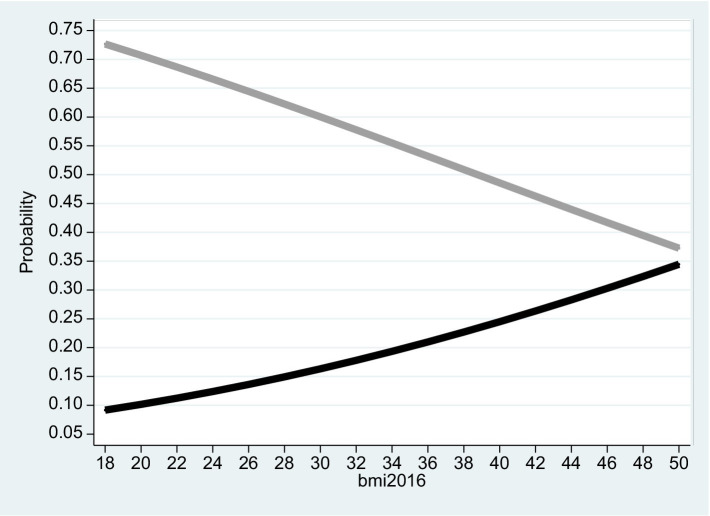
Females 2016. The figure is based on projected probabilities to report: “I often consider myself poor” (black line) and “I never consider myself poor” (grey line) as a function of the BMI level. The transformations to these projected probabilities are based on the outcomes reported on column (1) in Table 3. The horizontal axis is the BMI level on a scale of between 18 to 50. *BMI* (= $\frac{\mathrm{kg}.}{\mathrm{mete}r^{2}}\alpha$ where 18 ≤ *BMI* ≤ 25 is normal weight; 25 ≤ *BMI* < 30 is overweight; *BMI* ≥ 30 is considered obesity – see the links to the WHO websites in the reference list).

**Figure 4:**
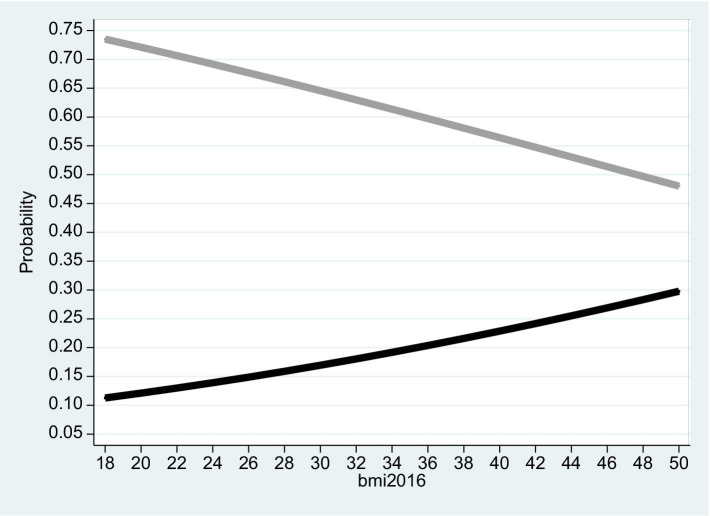
Males 2016. The figure is based on projected probabilities to report: “I often consider myself poor” (black line) and “I never consider myself poor” (grey line) as a function of the BMI level. The transformations to these projected probabilities are based on the outcomes reported on column (1) in Table 4. The horizontal axis is the BMI level on a scale of between 18 to 50. *BMI* (= $\frac{\mathrm{kg}.}{\mathrm{mete}r^{2}}$ where 18 ≤ *BMI* ≤ 25 is normal weight; 25 ≤ *BMI* < 30 is overweight; *BMI* ≥ 30 is considered obesity – see the links to the WHO websites in the reference list).

**Figure 5:**
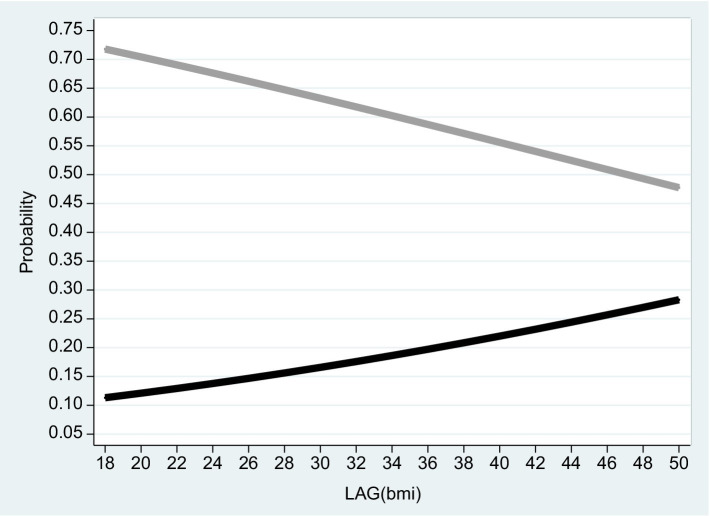
Pooled sample 2015–2016 (lagged variables). The figure is based on projected probabilities to report: “I often consider myself poor” (black line) and “I never consider myself poor” (grey line) as a function of the LAG (BMI) level. The transformations to these projected probabilities are based on the outcomes reported on column (1) in Table 5. The horizontal axis is the BMI level on a scale of between 18 to 50. *BMI* (= $\frac{\mathrm{kg}.}{\mathrm{mete}r^{2}}$ where 18 ≤ *BMI* ≤ 25 is normal weight; 25 ≤ *BMI* < 30 is overweight; *BMI* ≥ 30 is considered obesity – see the links to the WHO websites in the reference list).

**Figure 6:**
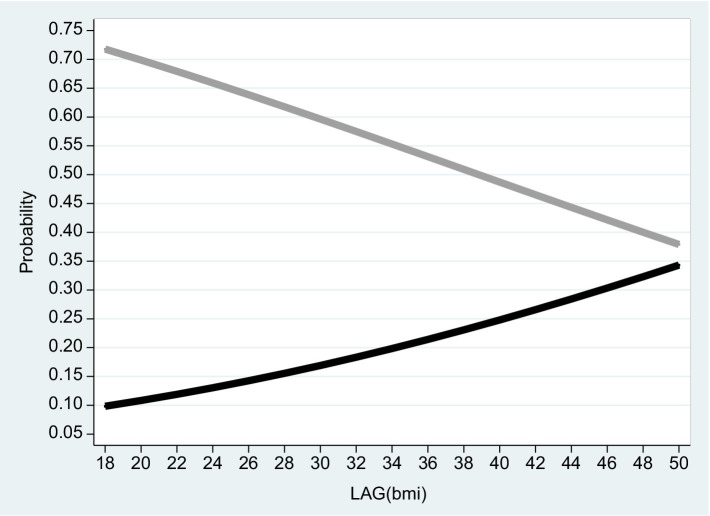
Females 2015–2016 (lagged variables). The figure is based on projected probabilities to report: “I often consider myself poor” (black line) and “I never consider myself poor” (grey line) as a function of the LAG(BMI) level. The transformations to these projected probabilities are based on the outcomes reported on column (1) in Table 6. The horizontal axis is the BMI level on a scale of between 18 to 50. *BMI* (= $\frac{\mathrm{kg}.}{\mathrm{mete}r^{2}}$ where 18 ≤ *BMI* ≤ 25 is normal weight; 25 ≤ *BMI* < 30 is overweight; *BMI* ≥ 30 is considered obesity – see the links to the WHO websites in the reference list).

**Figure 7:**
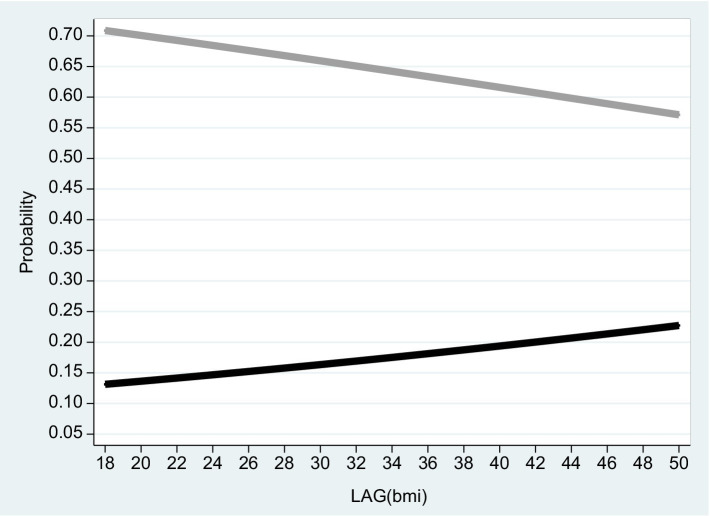
Males 2015–2016 (lagged variables). The figure is based on projected probabilities to report: “I often consider myself poor” (black line) and “I never consider myself poor” (grey line) as a function of the LAG(BMI) level. The transformations to these projected probabilities are based on the outcomes reported on column (1) in Table 7. The horizontal axis is the BMI level on a scale of between 18 to 50. *BMI* (= $\frac{\mathrm{kg}.}{\mathrm{mete}r^{2}}$ where 18 ≤ *BMI* ≤ 25 is normal weight; 25 ≤ *BMI* < 30 is overweight; *BMI* ≥ 30 is considered obesity – see the links to the WHO websites in the reference list).

The estimation results from this procedure are hard to interpret directly. Consequently, in subsequent sections, we provide for each Table the corresponding Figure, so that Figures 2–7 corresponds to column (1) in Tables 2–7. While Tables 2, 5 reports the outcomes obtained from the pooled sample, Tables 3, 4, 6, 7 report the outcomes obtained for the sub-sample of females and males.

**Table 2:** Survey data and ordered probit regressions: pooled sample.

	(1)	(2)
	SVY: ordered probit	Ordered probit
Variables	*Self_Rank_Poverty_*2016	*Self_Rank_Poverty_2016*
ln (Total_inc2016)	0.179*** (0.0167)	0.174*** (0.0168)
*BMI*2016	−0.0260*** (0.00454)	−0.0257*** (0.00450)
$Cut_{1}$ ( 1≤Self_Rank_Poverty_2016≤2 )	0.249 (0.212)	0.200 (0.216)
$Cut_{2}$ ( 2≤Self_Rank_Poverty_2016≤3 )	0.594*** (0.213)	0.546** (0.216)
$Cut_{3}$ ( 3≤Self_Rank_Poverty_2016≤4 )	0.902*** (0.213)	0.856*** (0.217)
Observations	4,017	4,017
Population	102,900.72	-
Strata	4	-

The estimation is based on the pooled sample of females and males obtained from the ICBS survey. The dependent variable (*Self_Rank_Poverty_2016*) is the self-ranking of individuals in response to the question: “During the last 15 years, how often did you consider yourself poor?” Possible answers are 1 = “often,” 2 = “sometimes,” 3 = “rarely,” 4 = “never.” The black (grey) line in the corresponding Figure 2 is the projected probability to answer 1 = “often” (4 = “never”) as a function of the BMI (= $\frac{\mathrm{kg}.}{{\mathrm{meter}}^{2}}$ where 18 ≤ BMI ≤ 25 is normal weight; 25 ≤ BMI < 30 is overweight; BMI ≥ 30 is considered obesity – see the links to the WHO websites in the reference list). Column (1) reports the outcomes of the survey data analysis. The analysis includes four strata based on the four categories of the variable *Self_Rank_Poverty_2016*. The weight given to each observation is based on the inverse of the BMI variable. Column (2) reports the outcomes where equal weight is given to each observation. In column (1)/(2) The linearized/conventional standard errors are given in parentheses ***p < 0.01.

**Table 3:** Survey data and ordered probit regressions: females.

	(1)	(2)
	SVY: ordered probit	Ordered probit
Variables	*Self_Rank_Poverty_*2016	*Self_Rank_Poverty_*2016
ln(Total_inc2016)	0.162*** (0.0255)	0.157*** (0.0256)
*BMI*2016	−0.0298*** (0.00661)	−0.0285*** (0.00660)
$Cut_{1}$ ( 1≤Self_Rank_Poverty_2016≤2 )	−0.0963 (0.325)	−0.122 (0.333)
$Cut_{2}$ ( 2≤Self_Rank_Poverty_2016≤3 )	0.287 (0.326)	0.267 (0.333)
$Cut_{3}$ ( 3≤Self_Rank_Poverty_2016≤4 )	0.634* (0.327)	0.615* (0.334)
Observations	1,788	1.788
Population	44,033.995	-
Strata	4	-

The estimation is based on the sample of females obtained from the ICBS survey. The dependent variable (*Self_Rank_Poverty_2016*) is the self-ranking of individuals in response to the question: “During the last 15 years, how often did you consider yourself poor?” Possible answers are 1 = “often,” 2 = “sometimes,” 3 = “rarely,” 4 = “never.” The black (grey) line in the corresponding Figure 3 is the projected probability to answer 1 = “often” (4 = “never”) as a function of the BMI (= $\frac{\mathrm{kg}.}{{\mathrm{meter}}^{2}}$ where 18 ≤ BMI ≤ 25 is normal weight; 25 ≤ BMI < 30 is overweight; BMI ≥ 30 is considered obesity – see the links to the WHO websites in the reference list). Column (1) reports the outcomes of the survey data analysis. The analysis includes four strata based on the four categories of the variable *Self_Rank_Poverty_2016*. The weight given to each observation is based on the inverse of the BMI variable. Column (2) reports the outcomes where equal weight is given to each observation. In column (1)/(2) The linearized/conventional standard errors are given in parentheses ***p < 0.01.

**Table 4:** Survey data and ordered probit regressions: males.

	(1)	(2)
	SVY: ordered probit	Ordered probit
Variables	*Self_Rank_Poverty_*2016	*Self_Rank_Poverty_*2016
ln(Total_inc2016)	0.193*** (0.0227)	0.189*** (0.0229)
*BMI*2016	−0.0224*** (0.00650)	−0.0228*** (0.00641)
$Cut_{1}$ ( 1≤Self_Rank_Poverty_2016≤2 )	0.550* (0.296)	0.498* (0.300)
$Cut_{2}$ ( 2≤Self_Rank_Poverty_2016≤3 )	0.868*** (0.297)	0.812*** (0.300)
$Cut_{3}$ ( 3≤Self_Rank_Poverty_2016≤4 )	1.146*** (0.298)	1.091*** (0.300)
Observations	2,229	2,229
Population	58,866.729	-
Strata	4	-

The estimation is based on the sample of males obtained from the ICBS survey. The dependent variable (*Self_Rank_Poverty_2016*) is the self-ranking of individuals in response to the question: “During the last 15 years, how often did you consider yourself poor?” Possible answers are 1 = “often,” 2 = “sometimes,” 3 = “rarely,” 4 = “never.” The black (grey) line in the corresponding Figure 4 is the projected probability to answer 1 = “often” (4 = “never”) as a function of the BMI (= $\frac{\mathrm{kg}.}{{\mathrm{meter}}^{2}}$ where 18 ≤ BMI ≤ 25 is normal weight; 25 ≤ BMI < 30 is overweight; BMI ≥ 30 is considered obesity – see the links to the WHO websites in the reference list). Column (1) reports the outcomes of the survey data analysis. The analysis includes four strata based on the four categories of the variable *Self_Rank_Poverty_2016*. The weight given to each observation is based on the inverse of the BMI variable. Column (2) reports the outcomes where equal weight is given to each observation. In column (1)/(2) The linearized/conventional standard errors are given in parentheses ***p < 0.01.

**Table 5:** Pooled sample 2015–2016 (lagged variables).

	(1)	(2)
	SVY: ordered probit	Ordered Probit
Variables	*Self_Rank_Poverty_*2016	*Self_Rank_Poverty_*2016
$\ln\left(\mathrm{LAG}\left(\mathrm{Total}\_inc\right)\right)$	0.152*** (0.0194)	0.148*** (0.0187)
*BMI*2016	−0.0221*** (0.00541)	−0.0208*** (0.00517)
$Cut_{1}$ ( 1≤Self_Rank_Poverty_2016≤2 )	0.0608 (0.244)	0.0456 (0.243)
$Cut_{2}$ ( 2≤Self_Rank_Poverty_2016≤3 )	0.376 (0.245)	0.366 (0.243)
$Cut_{3}$ ( 3≤Self_Rank_Poverty_2016≤4 )	0.695*** (0.246)	0.683*** (0.243)
Observations	3,083	3,083
Population	82,128.298	-
Strata	4	-

The estimation is based on the sample of females obtained from the ICBS survey. The dependent variable (*Self_Rank_Poverty_2016*) is the self-ranking of individuals in response to the question: “During the last 15 years, how often did you consider yourself poor?” Possible answers are 1 = “often,” 2 = “sometimes,” 3 = “rarely,” 4 = “never.” The black (grey) line in the corresponding Figure 5 is the projected probability to answer 1 = “often” (4 = “never”) as a function of the BMI (= $\frac{\mathrm{kg}.}{{\mathrm{meter}}^{2}}$ where 18 ≤ BMI ≤ 25 is normal weight; 25 ≤ BMI < 30 is overweight; BMI ≥ 30 is considered obesity – see the links to the WHO websites in the reference list). LAG(Total_inc) and LAG(BMI) are the Total Income and BMI reported in the 2015 wave. The objective of this exercise is to avoid endogeneity problems. Column (1) reports the outcomes of the survey data analysis. The analysis includes four strata based on the four categories of the variable *Self_Rank_Poverty_2016*. The weight given to each observation is based on the inverse of the LAG(BMI) variable. Column (2) reports the outcomes where equal weight is given to each observation. In column (1)/(2) The linearized/conventional standard errors are given in parentheses ***p < 0.01.

**Table 6:** Females 2015–2016 (lagged variables).

	(1)	(2)
	SVY: ordered probit	Ordered probit
Variables	*Self_Rank_Poverty_*2016	*Self_Rank_Poverty_*2016
$\ln\left(\mathrm{LAG}\left(\mathrm{Total}\_inc\right)\right)$	0.122*** (0.0284)	0.114*** (0.0284)
*BMI*2016	−0.0320*** (0.00771)	−0.0288*** (0.00748)
$Cut_{1}$ ( 1≤Self_Rank_Poverty_2016≤2 )	−0.570 (0.358)	−0.573 (0.367)
$Cut_{2}$ ( 2≤Self_Rank_Poverty_2016≤3 )	−0.206 (0.359)	−0.201 (0.367)
$Cut_{3}$ ( 3≤Self_Rank_Poverty_2016≤4 )	0.142 (0.359)	0.143 (0.367)
Observations	1,366	1,366
Population	33,771.645	-
Strata	4	-

The estimation is based on the sample of females obtained from the ICBS survey. The dependent variable (*Self_Rank_Poverty_2016*) is the self-ranking of individuals in response to the question: “During the last 15 years, how often did you consider yourself poor?” Possible answers are 1 = “often,” 2 = “sometimes,” 3 = “rarely,” 4 = “never.” The black (grey) line in the corresponding Figure 6 is the projected probability to answer 1 = “often” (4 = “never”) as a function of the BMI (= $\frac{\mathrm{kg}.}{{\mathrm{meter}}^{2}}$ where 18 ≤ BMI ≤ 25 is normal weight; 25 ≤ BMI < 30 is overweight; BMI ≥ 30 is considered obesity – see the links to the WHO websites in the reference list). LAG(Total_inc) and LAG(BMI) are the Total Income and BMI reported in the 2015 wave. The objective of this exercise is to avoid endogeneity problems. Column (1) reports the outcomes of the survey data analysis. The analysis includes four strata based on the four categories of the variable *Self_Rank_Poverty_2016*. The weight given to each observation is based on the inverse of the LAG(BMI) variable. Column (2) reports the outcomes where equal weight is given to each observation. In column (1)/(2) The linearized/conventional standard errors are given in parentheses ***p < 0.01.

**Table 7:** Males 2015–2016 (lagged variables).

	(1)	(2)
	SVY: ordered probit	Ordered Probit
Variables	*Self_Rank_Poverty_*2016	*Self_Rank_Poverty_*2016
$\ln\left(\mathrm{LAG}\left(\mathrm{Total}\_inc\right)\right)$	0.171*** (0.0273)	0.172*** (0.0255)
*BMI*2016	−0.0136* (0.00785)	−0.0138* (0.00745)
$Cut_{1}$ ( 1≤Self_Rank_Poverty_2016≤2 )	0.542 (0.354)	0.555 (0.345)
$Cut_{2}$ ( 2≤Self_Rank_Poverty_2016≤3 )	0.822** (0.355)	0.835** (0.345)
$Cut_{3}$ ( 3≤Self_Rank_Poverty_2016≤4 )	1.118*** (0.356)	1.132*** (0.345)
Observations	1,717	1,717
Population	45,429.417	-
Strata	4	-

The estimation is based on the sample of females obtained from the ICBS survey. The dependent variable (*Self_Rank_Poverty_2016*) is the self-ranking of individuals in response to the question: “During the last 15 years, how often did you consider yourself poor?” Possible answers are 1 = “often,” 2 = “sometimes,” 3 = “rarely,” 4 = “never.” The black (grey) line in the corresponding Figure 7 is the projected probability to answer 1 = “often” (4 = “never”) as a function of the BMI (= $\frac{\mathrm{kg}.}{{\mathrm{meter}}^{2}}$ where 18 ≤ BMI ≤ 25 is normal weight; 25 ≤ BMI < 30 is overweight; BMI ≥ 30 is considered obesity – see the links to the WHO websites in the reference list). LAG(Total_inc) and LAG(BMI) are the Total Income and BMI reported in the 2015 wave. The objective of this exercise is to avoid endogeneity problems. Column (1) reports the outcomes of the survey data analysis. The analysis includes four strata based on the four categories of the variable *Self_Rank_Poverty_2016*. The weight given to each observation is based on the inverse of the LAG(BMI) variable. Column (2) reports the outcomes where equal weight is given to each observation. In column (1)/(2) The linearized/conventional standard errors are given in parentheses ***p < 0.01.

In each of the subsequent sections, column (1) of each Table reports the outcomes of the survey data analysis. The analysis includes four strata based on the four categories of the variable *Self_Rank_Poverty_*2016. The weight given to each observation is based on the inverse of the BMI variable. Column (2) reports the outcomes where equal weight is given to each observation.

The remainder of the manuscript consists of two parts:

The basic results part refers to a cross section obtained from the 2016 wave. The dependent variable is *Self_Rank_Povery_*2016, the self-ranking of individuals in response to the question: “During the last 15 years, how often did you consider yourself poor?” Possible answers are 1 = “often,” 2 = “sometimes,” 3 = “rarely,” 4 = “never”; The independent variables are ln (Total_Inc2016) (the natural logarithm of the total income); and BMI2016 (= $\frac{\mathrm{kg}.}{\mathrm{mete}r^{2}}$ where 18 ≤ *BMI* ≤ 25 is normal weight; 25 ≤ *BMI* < 30 is overweight; *BMI* ≥ 30 is considered obesity – see the links to the WHO websites in the reference list).

An important concern is the potential endogeneity problem between Total_Inc2016 / BMI2016 and *Self_Rank_Povery*_2016. Differently put, the chicken and egg problem might arise: Does reduced ranking motivate weight gain or vice versa (weight gain motivates lower ranking). The robustness test part investigates this problem by replacing the independent variables ln(Total_Inc2016) and BMI2016 by ln(LAG(Total_Inc)) = ln(Total_Inc2015) and LAG(BMI) = BMI2015.

Finally, we run the Granger Causality test separately for females and males. This test permit testing whether lagged BMI Granger-cause different self-ranking of poverty.

## 3 Results

### 3.1 Basic results

Based on the empirical model, Figures 2–4 correspond to Tables 2–4 and describe the projected probability to often (never) consider yourself poor as a function of the *BMI* when income is controlled. While Figure 2 refers to the pooled sample, Figures 3, 4 are stratified by gender (females and males).

The Tables exhibit the “correct” signs of the two estimated coefficients of the independent variables. The sign of the ln(Total_Inc_2016) coefficient is positive and significant. Given that a *rise* in *Self_Rank_Poverty_2016* (the dependent variable) is associated with reduced subjective sense of poverty, the implication is that when income level *rise* and the model is BMI adjusted, the inclination to rank yourself as “poor” *drops*. In contrast, the sign of the *BMI*_2016 coefficient is negative and significant. The implication is that when the *BMI* variable *increases* and the model is income adjusted, the inclination to rank yourself as “poor” *rises*.

It is evident from the three figures that for the same level of income and for both genders, the projected probability of self-ranking as “not poor” *drops* from 0.73 where the *BMI* equals 18 to 0.37 (females) – 0.48 (males) where the *BMI* equals 50. At the same time, the projected probability of self-ranking as “poor” *rises* from 0.09 (females) – 0.11 (males) where the *BMI* equals 18 to 0.34 (females) – 0.30 (males) where the *BMI* equals 50. Unlike Arbel et al. (6), no gender differences were recorded in this section.

### 3.2 Robustness tests

To address the potential endogeneity problem between the *BMI* and *Self_Rank_Poverty_*2016, we ran the same model based on a follow-up of two years (2015 and 2016), where the independent variables are the lagged *BMI* and the natural logarithm of lagged total income from all sources. This ensures that the independent variables are exogenous. The outcomes, given in Tables 5–7 and the corresponding Figures 5–7, are robust to those obtained previously.

Finally, to investigate the casual relationships between the ranking of poverty and the independent variables, we ran the Granger causality test for the pooled sample, and separately for females and males [(31, 32): 476–477]. A detailed description of the test is given in Appendix B. Results of this test are reported in Table 8.

**Table 8:** Granger causality test.

	Calculated	Calculated *p*-value
Pooled Sample	*F*(1, 4,661) = 6.52	*p* = 0.0107
Females	*F*(1, 2,311) = 6.62	*p* = 0.0102
Males	*F*(1, 2,344) = 1.33	*p* = 0.2491

The calculated F-statistics of the granger causality test is given by: $F_{C}=\frac{\left(ESS_{R}-ESS_{U}\right)/1}{ESS_{U}/\left(N-2\right)}$ . Where $ESS_{R}$ $\left(ESS_{U}\right)$ is the error sum of square of the restricted (unrestricted) model, and N is the number of observations. The unrestricted model is given by: $Avg\_\mathrm{Self}\_\mathrm{Rank}\_\mathrm{Poverty}=\alpha_{1}\mathrm{LAG}\left(Avg\_\mathrm{Self}\_\mathrm{Rank}\_\mathrm{Poverty}\right)+\alpha_{2}\mathrm{LAG}\left(Avg\_BMI\right)+\mu_{1}$ . And the restricted model is given by: $Avg\_\mathrm{Self}\_\mathrm{Rank}\_\mathrm{Poverty}=\beta_{1}\mathrm{LAG}\left(Avg\_\mathrm{Self}\_\mathrm{Rank}\_\mathrm{Poverty}\right)+\mu_{2}$ . Where each variable is manifested in terms of the difference from the respective mean; Avg_Wealth Avg_Bmi are the differences between the wealth divided by four (to scale the ordinal outcome between 0 and 1); the BMI and their respective means; the LAG operator is the variable lagged by one year; $\alpha_{1},\alpha_{2},\beta_{1}$ are estimated parameters; and $\mu_{1},\mu_{2}$ are the classical random disturbance term.

The outcomes demonstrate that for women, the *BMI* Granger-cause the poverty subjective ranking at the 5% level. Yet, for the male group, there are no casual relationships between the *BMI* as a proxy for obesity and self-ranking of poverty. Several studies demonstrate that compared to men, women are penalized more severely due to obesity, including in prospects of finding employment [e.g., (12, 17, 33–42)]. In this context, Arbel et al. (7) demonstrate that compared to men, female self-evaluation of housing prices is more conservative and less influenced by *BMI* changes. This outcome is obtained despite the fact that women are more susceptible to weight gain, particularly in western societies [e.g., (18, 43, 44)].

To further explore the external validity, we employ an approach based on Friedman (45) and machine learning (46), consisting of the following steps:

1. the cross-validation command in Stata. The command: (a) randomly assigns the sample to training (on-sample) group and test (off-sample) group and (b) generates a vector of predictions on the test group based on the outcomes obtained from the training group (Proj1).
2. Post-estimation results of projections obtained when the pooled sample is employed (Proj_ *Self_Rank_Poverty*).
3. Regression analysis between the vector of predictions obtained from step 1 and step 2.
4. If external validity exists, the null hypothesis of no constant and a slope of one (a $45^{{}^{\circ}}$ angle) should not be rejected.
5. The outcomes of this procedure are given in Table 9. They indeed demonstrate that the null hypothesis of no constant and a slope of one cannot be rejected for both genders at the 1% significance level (*p* = 0.0167–0.0195).

**Table 9:** External validity test.

	Women	Men
Variables	*Proj_Self_Rank_Poverty_*2016	*Proj_Self_Rank_Poverty_*2016
Constant	0.0004 (0.411)	0.0012 (0.158)
Proj1	0.9942*** (<0.01)	0.9885*** (<0.01)
Observations	1,788	2,229
F-test		
*F*-value (Const = 0; coef(Proj1)) = 1	4.10	3.94
d.f. numerator	2	2
d.f. denominator	1,786	2,227
*P*-value (Const = 0; coef(Proj1)) = 1	0.0167	0.0195

*p*-values are given in parentheses. ***p < 0.01.

## 4 Discussion

This study introduces a novel approach to examining the impact of societal norms on obese women, focusing on their economic self-perception. By controlling for income levels over a two-year period, the research reveals a significant decrease in the likelihood of obese individuals ranking themselves as “not poor,” particularly among women (from 0.73 to 0.37), and men (0.48). This underscores how weight biases influence economic self-assessment, independent of actual income variations.

Moreover, the study contributes to understanding the concept of permanent income among obese individuals, highlighting a discrepancy between their current and expected long-term income levels. Unlike prior research focusing mainly on self-esteem impacts, this study reveals a heightened perception of poverty among obese persons, indicating broader societal implications beyond health outcomes.

The findings also suggest a gender-specific effect, where lagged BMI significantly predicts self-ranking of poverty among women but not men, suggesting differential economic impacts based on weight.

Public policy implications are substantial, advocating for interventions to mitigate weight-based discrimination in employment and social settings. Efforts to enhance self-esteem and economic opportunities for obese individuals, similar to campaigns for other marginalized groups, are recommended.

Research findings support the awareness of more obese women, manifested by their subjective ranking as “not poor,” to lower prospects of finding a job.

This support is consistent with the results demonstrating a *drop* in the projected probability of self-ranking as “not poor” with the *BMI* from 0.73 to 0.37 among females. Differently put, the study reveals the inclination of more obese women to rank themselves as “poor” and less obese women to rank themselves as “not poor.” Given that the income level is controlled, under equal conditions, higher BMI will elevate the women’s tendency to feel poorer compared to their less obese counterparts.

Research findings thus stress the need to educate the public against prejudices on the grounds of obesity, particularly given that 0.78–0.81 of the weight gain is attributed to heritability (24). In that context, Shugart (47) demonstrate a shift in the American public opinion following *The Oprah Winfrey Show,* from an historical attribution of obesity to personal responsibility to cultural explanations. Ophra Winfrey is known for her own public struggle with obesity, which she often engages in on her show. This serves further to anchor the moral authority on the topic and the reflection of the fact that obesity is not necessarily the outcome of lack of will power. Consequently, there is no reason why employers should offer obese women less job opportunities compared to their normal weight counterparts [e.g., (17)].

In particular, our study seeks to evoke awareness among potential employers, which, in turn, might motivate avoidance of, or at least reduction in, an implicit wage penalty against obese women (to which the women are aware of according to a possible interpretation of our findings).

This conclusion is further supported by Ásgeirsdóttir et al. (8), Chung and Lim (48), and Arbel et al. (6, 7).

Ásgeirsdóttir et al. (8) suggest that only females show a positive willingness to pay (WTP) for not being overweight. Based on their income level, and to achieve the same happiness level, the reduction of weight for overweight females is associated with WTP of $3,608–$37,488 per year.

Based on Korea National Health and Nutrition Examination Survey (2010–2014), Chung and Lim (48) found that obesity prevalence more than doubled with a shift from less to more educated women. While 34.3% of the less-educated women were defined “obese,” this prevalence reduced to only 16.0% among the highly educated women. Given the return on higher human capital, higher education is also positively associated with income level. In this context Mathieu-Bolh (49) suggests that among socio-economic characteristics poverty seems to be connected to obesity in rich countries, albeit this relationship might be more elusive than expected.

Arbel et al. (6) demonstrate another aspect of the penalty against obese women: their need to compromise on men with shorter height as mates. The literature demonstrates positive association between the height of the men, and owning a car, having more children, and living in a single family detached unit. Stefanczyk et al. (50), for instance, showed that shorter men are rated by others as less masculine, less physically attractive, of lower social and professional status, and less competent compared with taller men.

Finally, Arbel et al. (7) explored the relationship between self-evaluation of apartments and obesity as a proxy for self-esteem, particularly among women. One would anticipate a lower self-evaluation of apartment value among obese women following the influence of western values and norms regarding a slim body image of women, namely, social obesity penalties. The authors demonstrate that for both genders, *BMI* is negatively correlated with self-evaluation of apartments. Yet, compared to males, the cognitive error in price evaluation is smaller among women.

## 5 Summary and conclusions

The current study proposes and applies a new measure for the influence of western social values and norms associated with discrimination against obese women. Based on a follow-up survey of two years, we estimate the relationship between projected probability of self-ranking of poor individuals and obesity, when the income level is controlled. Findings clearly demonstrate a *drop* the projected probability of self-ranking as “not poor” with *BMI* from 0.73 to 0.37 (females) – 0.48 (males) when income is controlled. Similar outcomes are obtained when the independent variables are lagged and thus avoid endogeneity concerns. Finally, additional outcomes support the conclusion that the lagged *BMI* Granger-cause self-ranking of poverty for women, but not for men.

The contribution of this manuscript lies in the new method proposed to assess the permanent income of obese persons. According to Friedman (23) the permanent income hypothesis is a theory of consumer spending stating that people will spend money at a level consistent with their expected long-term average income. The outcomes of the current study indicate that the level of permanent income among obese person is lower than their current income.

Unlike previous studies, our manuscript shows that obese persons suffer not only from poor self-esteem (4, 5, 22), but also from an increased sense of poverty among both women and men even, when the actual level of income is controlled. In response to the question—how often do you consider yourself poor, a higher inclination among obese person to answer “often” was observed. Unlike Böckerman et al. (20), who demonstrate the impact of genetic factors, the current manuscript exemplifies the cultural factors of a social stigma in our thin-obsessed society (22).

The outcomes of our research may be interpreted as the degree of awareness to the social stigma concerning obese persons among potential employers, parents, and mates. This pattern, in turn, adversely affects the Social and Economic Status (SES) in the long run. In this context, a recent article by Campos-Vazquez and Gonzalez (17) indeed demonstrated a lower prevalence of job offers to obese women in Mexico– where all the other C.V. factors were controlled.

Discrimination and prejudice may also be considered as irrational behavior. Preston and Szymanski (51) demonstrated that increasing the proportion of black soccer players is expected to improve the ranking of English football teams. Goldin and Rouse (52) provide evidence suggesting that the blind audition procedure fostered impartial hiring of musicians and increased the proportion of women in symphony orchestras.

The public policy repercussions of our study should be divided into two main components. The first relates to the work of professionals (psychologists, doctors, dietitians, personal trainers and social workers) with obese persons, in an effort to boost their self-esteem, provide motivation for development and assist them in maintaining appropriate nutrition and participating in physical activity programs. Undoubtedly, obesity is a medical problem and the risk factor of many diseases such as cardiovascular diseases (mainly heart disease and stroke), which were the leading cause of death in 2012; diabetes; musculoskeletal disorders (especially osteoarthritis – a highly disabling degenerative disease of the joints); several cancer types (including endometrial, breast, ovarian, prostate, liver, gallbladder, kidney, and colon) (53, 54). Solely from health considerations, obese persons should be monitored and encouraged to reduce weight.

The second component is the need for a broad-based public information campaign, explaining the genetic sources of obesity. About 60–80% of obesity and severe obesity might be explained by genetic factors (55, 56). Indeed, Shugart (47) demonstrate a shift in the American public opinion following *The Oprah Winfrey Show,* from an historical attribution of obesity to personal responsibility to cultural explanations. Ophra Winfrey is known for her own public struggle with obesity, which she often engages on her show. This serves further to anchor the moral authority on the topic and the reflection of the fact that obesity is not necessarily the outcome of lack of will power. Consequently, there is no reason why employers should offer obese women less job opportunities compared to their normal weight counterparts [e.g., (17)].

The strength of our study may be described as follows:

1. The potential relationship between obesity and self-ranking of poverty has not been previously explored based on self-ranking of poverty when income is controlled. We thus propose a new measure for the influence of western social values and norms associated with discrimination against obese women.
2. Unlike previous studies, our manuscript shows that obese persons suffer not only from poor self-esteem (4, 5, 22), but also from an increased sense of poverty among both women and men even, when the actual level of income is controlled.
3. In particular, our study seeks to evoke awareness among potential employers, which, in turn, might motivate avoidance of, or at least reduction in, an implicit wage penalty against obese women.

There are several weaknesses associated with this study. The study uses BMI as a measure of obesity. According to the World Health Organization, on the one hand, like any other measure BMI is not perfect indicator since it is only dependent on height and weight and it does not take into consideration different levels of adiposity based on age, physical activity levels and gender. For this reason it is expected that it overestimates adiposity in some cases and underestimates it in others (53).

On the other hand, BMI is easy to measure and calculate and is therefore the most commonly used tool to correlate risk of health problems with the weight at population level. During the 1970s and based especially on the data and report from the Seven Countries study, researchers noticed that BMI appeared to be a good proxy for adiposity and overweight related problems (53).

Other measures of obesity are also problematic. Association between waist circumferences (WC) and health risks is not a trivial exercise and should be undertaken scientifically using proper techniques (53).

A further discussion of the advantages and disadvantages of BMI and other possible measures of obesity are given in Nirav and Braverman (57), Antonopoulos et al. (58), Bosello et al. (59), Gažarová et al. (60), and Moltrer et al. (61).

Another limitation that should be considered is the self-reported BMI in our sample. Yet, two points should be considered:

1. The survey is an interview, so that the interviewer can get an impression whether the self-report is reasonable.
2. The figure given in Appendix C provides the prevalence of obesity based measured vs. self-reported BMI in OECD countries. As can be seen in this figure, unlike countries like Chile, Portugal, Australia, Turkey, Hungary and Ireland, in Israel there is almost no difference between the prevalence of obesity based measured vs. self-reported BMI.

Finally, an additional limitation is the possible extension of the proposed empirical model which will considers the potential non-linear effect of obesity. This is a subject for possible future research. Such research should employ parabolic models, which permit non-monotonic increase or decrease with obesity.

## Data Availability

The original contributions presented in the study are included in the article/Supplementary material, further inquiries can be directed to the corresponding author.

## Footnotes

^1^It should be noted, however, that according to Böckerman et al. (20), the use of a newer, broader genetic risk score, changes this outcome to non-rejection of the null hypothesis of no effect of obesity on these factors.

^2^BMI is formerly called the Quetelet index (named after the 19th century Belgian scientist – Adolf Quetelet – who was the first to formulate this measure). According to the World Health Organization (WHO) definitions (53, 54), 18 ≤ *BMI* < 25 is considered normal weight; 25 ≤ *BMI* ≤ 30 is considered overweight, and *BMI* > 30 is considered obesity – see https://www.euro.who.int/en/health-topics/disease-prevention/nutrition/a-healthy-lifestyle/body-mass-index-bmi and https://www.who.int/news-room/fact-sheets/detail/obesity-and-overweight.

^3^In this context, Stunkard et al. (56) suggested that: “Classic twin methods estimated a high heritability for height, weight, and BMI, both at age 20 years (0.80, 0.78, and.77, respectively) and at a 25-year follow-up (0.80, 0.81, and.84, respectively). Height, weight, and BMI were highly correlated across time, and a path analysis suggested that the major part of that covariation was genetic. These results are similar to those of other twin studies of these measures and suggest that human fatness is under substantial genetic control.” (the Abstract)

^4^Referring separately to the 2015 wave, and to the 2015–2016 waves without lagged explanatory variables, results still remain robust. These complementary outcomes are available upon request.

^5^Note the difference between the sample and population income. In the sample, the variable Total_inc refers to income from all sources (including self-employment, wage, royalties, babysitting and all other sources). In the population, the 121,476 NIS figure refers to annual income only from wage.

^6^Sen (62) criticized the conventional measures of poverty. Those are based either on the percent of households whose net income is below the poverty line *z* ( $H=\frac{q}{N}$ where *q* is the number of households whose income is below *z* and *N* is the total population) or by measuring the aggregated difference between the net income of the poor to the poverty line ( $I=\sum \frac{g_{i}}{qz}$ where $g_{i}$ is the difference for individual *i*). Sen (62) proposed a new measure based on the formula $P=H\left[I+\left(1-I\right)G\right]$ where *G* is the Gini coefficient.

^7^To avoid outliers, the model incorporates the logarithmic transformation of the variable Total_Inc2016.
