# Supplementary material for: Does obesity create a relative sense of excess poverty?
Source: Front Public Health. 2024 Nov 27;12:1480365. doi: 10.3389/fpubh.2024.1480365 (PMC11633321; doi:10.3389/fpubh.2024.1480365)
Supplement: Supplementary file 1 [file Data_Sheet_1.pdf]

Appendix A: Detailed Description of the Ordered Probit Regression

This study employs the ordered probit methodology, which is based on the following non-linear formula of the cumulative normal distribution function: $\int_{-\infty}^{\infty} \frac{e^{-(x-\mu)^2/2\sigma^2}}{\sqrt{2\pi\sigma^2}} dx$.[1]

Following Johnston and Dinardo (1997 [41]) and Frey & Stutzer (2002 [27]) we define the following ordered probit empirical model:

This model is designed to exploit the ordering information, where category i = 1 (the subject often considers himself or herself poor) is defined as the minimum value of the variable, i = 2 (the subject sometimes considers himself or herself poor) as the next ordered value, and so on, for the empirically determined i=4 categories (the subject never considers himself or herself poor).

The projected probability of switching from one category to the subsequent one (p_{ij}) for category i and subject j is given by the following equation:

$$p_{ij} = Pr(y_j = i) = Pr(k_{i-1} < x_j\beta + u \le k_i) = \Phi(k_i - x_j\beta) - \Phi(k_{i-1} - x_j\beta) \quad (1)$$

Where k_i is the ordinal number for category i, k_0 is defined as $-\infty$, k_5 is defined as $+\infty$, $y_j = wealth$; $\Phi(\cdot)$ is the cumulative normal distribution function, x_j is a matrix of j individuals (rows) and two independent variables (columns of $x_j = (BMI2016;\ ln(Total\_INC2016))$).

More specifically to our case, the model may be defined as follows:

$$prob(1 \le Self\_Rank\_Poverty\_2016 \le 2) = F(cut_1 - \alpha_1 Total\_Inc2016 - \alpha_2 BMI2016)$$

[1] Information about this methodology is based on the Stata 16.1 manual. According to Frey & Stutzer (2002 [27]): "Provided that reported subjective well-being is a valid and empirically adequate measure for human well-being, it can be modeled in a microeconometric happiness function $W_{it} = \alpha + \beta X_{it} + \epsilon_{it}$ that is estimated by ordered probit or logit." (page 406).

$prob(2 \leq Self\_Rank\_Poverty\_2016 \leq 3) = F(cut_2 - \alpha_1 ln(Total\_Inc2016) - \alpha_2 BMI2016) - F(cut_1 - \alpha_1 ln(Total\_Inc2016) - \alpha_2 BMI2016)$

$prob(3 \leq Self\_Rank\_Poverty\_2016 \leq 4) = F(cut_3 - \alpha_1 ln(Total\_Inc2016) - \alpha_2 BMI2016) - F(cut_2 - \alpha_1 ln(Total\_Inc2016) - \alpha_2 BMI2016) - F(cut_1 - \alpha_1 ln(Total\_Inc2016) - \alpha_2 BMI2016)$

$prob(Self\_Rank\_Poverty\_2016 = 1) = 1 - F(cut_3 - \alpha_1 ln(Total\_Inc2016) - \alpha_2 BMI2016) - F(cut_2 - \alpha_1 ln(Total\_Inc2016) - \alpha_2 BMi2016) - F(cut_1 - \alpha_1 ln(Total\_Inc2016) - \alpha_2 BMI2016)$

Where $Self\_Rank\_Poverty\_2016$ is the self-ranking of individuals in response to the question: "During the last 15 years, how often did you consider yourself poor?" Possible answers are 1="often", 2="sometimes", 3="rarely", 4="never"; the independent variables are $ln(Total\_Inc2016)$ and $BMI2016$

The estimated log likelihood function is given by the following equation:

$$lnL = \sum_{j=1}^{N} \omega_j \sum_{i=1}^{4} I_i(y_j) lnp_{ij} \quad (2)$$

Where ln$L$ is the natural logarithm of the log-likelihood function; ω_j is the optional weight (the default is $\omega_j = \frac{1}{N}$ where N is the number of subjects); and $I_i(y_j) = \begin{cases} 1, & if\ y_j = i \\ 0, & otherwise \end{cases}$ outcomes also give three cutoff points (one for each shift from one category to another).
