# Supplementary material for: Does obesity create a relative sense of excess poverty?
Source: Front Public Health. 2024 Nov 27;12:1480365. doi: 10.3389/fpubh.2024.1480365 (PMC11633321; doi:10.3389/fpubh.2024.1480365)
Supplement: Supplementary file 2 [file Data_Sheet_2.pdf]

Appendix B: Detailed Description of the Granger Causality Test

The Granger causality test for the pooled sample, and separately for females and male is based on Granger, 1969 [33] and is described inter-alia in Ramanathan, 2002 [52]: 476-47.

The unrestricted model of the Granger causality test (adapted to our study) is given by:

$$Avg\_Self\_Rank\_Poverty = \alpha_1 LAG(Avg\_Self\_Rank\_Poverty) + \alpha_2 LAG(Avg\_BMI) + \mu_1$$

And the restricted model is given by:

$$Avg\_Self\_Rank\_Poverty = \beta_1 LAG(Avg\_Self\_Rank\_Poverty) + \mu_2$$

Where each variable is manifested in terms of difference from the respective mean; $Avg\_Self\_Rank\_Poverty\_2016$ Avg_BMI are the differences between the *Self_Rank_Poverty_*2016 divided by four (to scale the ordinal outcome between 0 and 1); the *BMI* and their respective means; the *LAG* operator is the variable lagged by one year; $\alpha_1, \alpha_2, \beta_1$ are estimated parameters; and μ_1, μ_2 are the classical random disturbance term. The calculated *F*-statistics of the Granger causality test are given by:

$$F_C = \frac{(ESS_R - ESS_U)/1}{ESS_U/(N-2)}$$

Where ESS_R (ESS_U) is the error sum of square of the restricted (unrestricted) model, and *N* is the number of observations.
