# Supplementary material for: Does obesity create a relative sense of excess poverty?
Source: Front Public Health. 2024 Nov 27;12:1480365. doi: 10.3389/fpubh.2024.1480365 (PMC11633321; doi:10.3389/fpubh.2024.1480365)
Supplement: Supplementary file 3 [file Data_Sheet_3.pdf]

Appendix C: Overweight or Obese Population (Measured/Self-Reported, % of Population Aged 15+, 2018 or Latest Available)

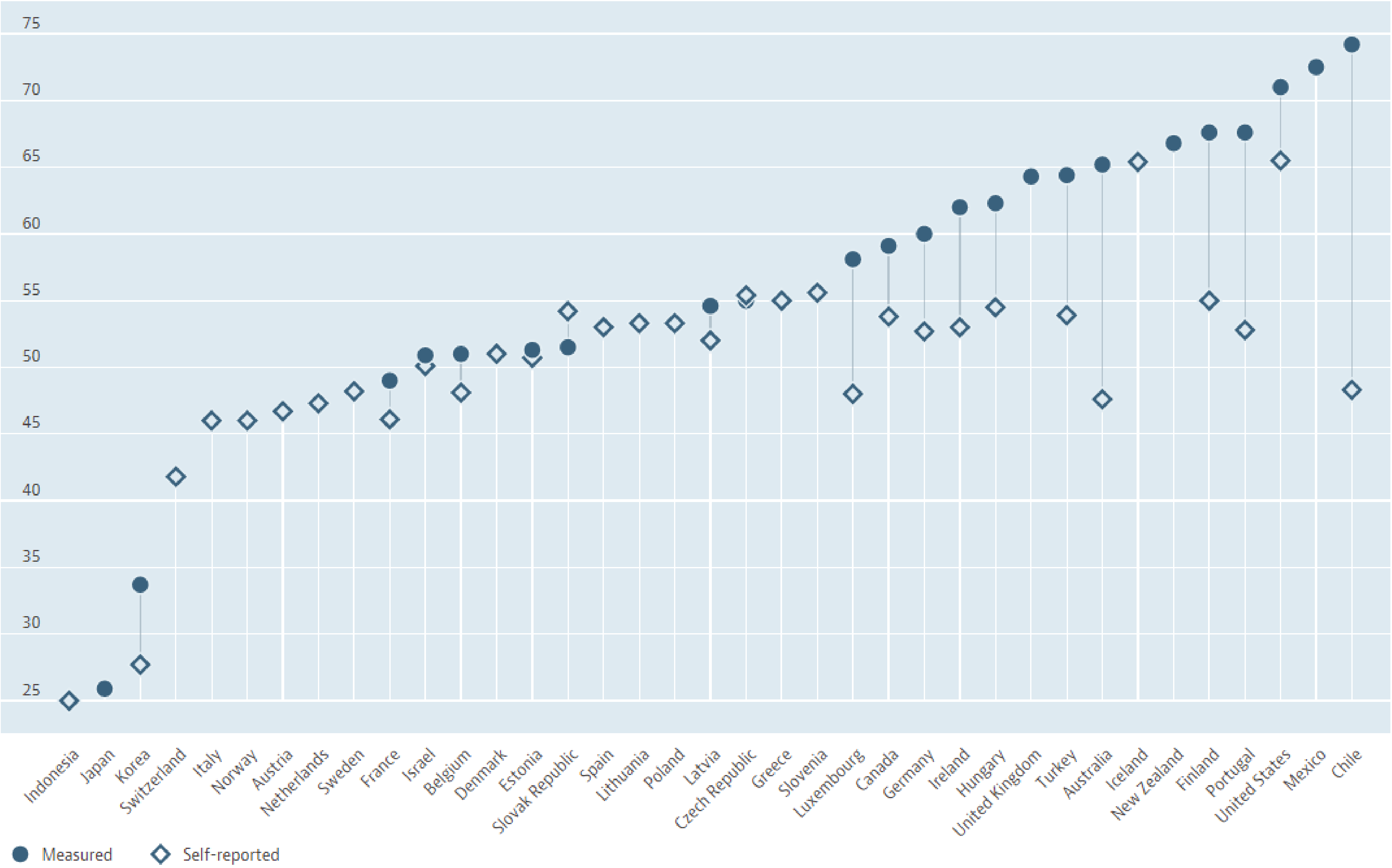

Source: OECD Report available at: https://data.oecd.org/pinboard-editor/ (Last accessed on June 30, 2021). Overweight is defined as: $25 \leq BMI < 30$ and obesity is defined as $BMI \geq 30$, where $BMI = \frac{kg}{meter^2}$.
